# The influence of cardiac output on propofol and fentanyl pharmacokinetics and pharmacodynamics in patients undergoing abdominal aortic surgery

**DOI:** 10.1007/s10928-020-09712-1

**Published:** 2020-08-25

**Authors:** Agnieszka Bienert, Paweł Sobczyński, Katarzyna Młodawska, Roma Hartmann-Sobczyńska, Edmund Grześkowiak, Paweł Wiczling

**Affiliations:** 1 Department of Clinical Pharmacy and Biopharmacy, Poznan University of Medical Sciences, Sw. Marii Magdaleny 14 Street, 61-861 Poznan, Poland; 2 Department of Anesthesiology and Intensive Therapy, Poznan University of Medical Sciences, 1/2 Długa Str., 61-848 Poznań, Poland; 3 Department of Experimental Anaesthesiology, Poznan University of Medical Sciences, Sw. Marii Magdaleny 14 Street, 61-861 Poznan, Poland; 4 Department of Biopharmacy and Pharmacodynamics, Medical University of Gdansk, Hallera 107 Street, 80-416 Gdansk, Poland

**Keywords:** Propofol, Fentanyl, Cardiac output, Pharmacokinetics, Pharmacodynamics

## Abstract

Cardiac output (CO) is expected to affect elimination and distribution of highly extracted and perfusion rate-limited drugs. This work was undertaken to quantify the effect of CO measured by the pulse pressure method on pharmacokinetics and pharmacodynamics of propofol and fentanyl administrated during total intravenous anesthesia (TIVA). The data were obtained from 22 ASA III patients undergoing abdominal aortic surgery. Propofol was administered via target-controlled infusion system (Diprifusor) and fentanyl was administered at a dose of 2–3 µg/kg each time analgesia appeared to be inadequate. Hemodynamic measurements as well as bispectral index were monitored and recorded throughout the surgery. Data analysis was performed by using a non-linear mixed-effect population modeling (NONMEM 7.4 software). Three compartment models that incorporated blood flows as parameters were used to describe propofol and fentanyl pharmacokinetics. The delay of the anesthetic effect, with respect to plasma concentrations, was described using a biophase (effect) compartment. The bispectral index was linked to the propofol and fentanyl effect site concentrations through a synergistic *E*_*max*_ model. An empirical linear model was used to describe CO changes observed during the surgery. Cardiac output was identified as an important predictor of propofol and fentanyl pharmacokinetics. Consequently, it affected the depth of anesthesia and the recovery time after propofol-fentanyl TIVA infusion cessation. The model predicted (not observed) CO values correlated best with measured responses. Patients‘ age was identified as a covariate affecting the rate of CO changes during the anesthesia leading to age-related difference in individual patient’s responses to both drugs.

**Electronic supplementary material:**

The online version of this article (10.1007/s10928-020-09712-1) contains supplementary material, which is available to authorized users.

## Introduction

Providing an adequate level of anaesthesia is challenging and requires careful monitoring and dose titration. Especially in the light of recent studies which have suggested that “too deep” anesthesia may increase long-term postoperative mortality in cardiac surgery patients [1, 2]. Therefore understanding the pharmacokinetics and pharmacodynamics (PK/PD) of drugs used in anesthesia is crucial, especially for rarely studied groups of patients or conditions.

Propofol is a short-acting hypnotic widely used for induction and maintenance of general anesthesia as well as for postoperative sedation in patients undergoing abdominal aortic surgery [3–5]. Different pharmacokinetic (PK) models of propofol have been presented for healthy patients, critically ill patients, as well as for animals [5–16]. The 3-compartmental models published by Marsh [15] and Schnider [16] are incorporated into the target-controlled infusion system (TCI) and serve as a guide for propofol administration. These models were developed based on healthy adults without any additional drug co-administrated. However, propofol is usually combined with an opioid drug to ensure adequate analgesia during total intravenous anesthesia. As a consequence various PK/PD interactions can occur [17–19]. For molecules with high hepatic extraction ratio, such as propofol and fentanyl, it is expected that changes in cardiac output (CO) influence their elimination clearance by affecting liver blood flow. Also, the distribution rate of drugs exhibiting perfusion rate-limited distribution, such as propofol and fentanyl, is expected to be affected by tissue blood flow [13, 20–22]. This mechanism was confirmed for several drugs used in anesthesia [20, 21, 23] and for some of them the impact of cardiac output values on the adequate dosing scheme of anesthetic drugs was confirmed under experimental conditions^21^. However, there is still very little clinical data to support dose-adjustments based on CO measurements. Upton et al^20^ have shown that after a short infusion of propofol, its initial concentrations are determined by cardiac output. On the other hand, in a pilot study by Peeters et al. [4] no significant relationship between measured CO and propofol clearance in ICU patients was observed. Therefore, it is still an open question as to whether CO may really by useful to predict the concentrations of propofol or fentanyl. Cardiovascular surgery ensures specific conditions which may be useful to study the influence of CO on propofol/fentanyl PK/PD, as it is characterized by changes in CO [24–27]. Today, the minimally invasive methods of the cardiac output monitoring have become more popular in the clinical practice [28] especially during cardiovascular surgeries, thus continuous CO monitoring might be useful in understanding PK/PD of propofol and fentanyl and consequently in guiding adequate drugs’ dosing.

The aim of this work was to build a PK/PD model for propofol-fentanyl TIVA in patients undergoing vascular surgeries. The model included CO as a dependent variable and included a relationship between CO and the PK parameters of both drugs. Further we examined whether CO measurements by minimally invasive pulse pressure method could be useful for clinical decision regarding the propofol and fentanyl dosing.

## Methods

### Patients

After the approval from the local Research Ethics Committee and written informed consent, 22 patients undergoing major aortic surgery, classified as ASA III according to the American Society of Anesthesiologists (ASA) physical status classification system, were enrolled in the study. We analyzed data collected in two studies in which propofol-opioid TIVA was used during major aortic surgery. In both of them CO was continuously measured during anesthesia and the same surgical and anesthetic procedures were applied as well. The only difference was related to more frequent measurement of CO, as well as propofol and fentanyl concentrations in the second study. The data from Study 1 were obtained from a previous publication published by our group^24^, nevertheless due to sparse data, we were unable to assess the influence of CO on the PK/PD of propofol and fentanyl. As clinical conditions of the studies were identical, we pooled all the data to better characterize propofol and fentanyl pharmacokinetics in these patients.

The exclusion criteria in both studies were: previous cardiac surgery, ejection fraction < 40%, valvular heart disease and myocardial infarction within 3 months prior to surgery, significant renal (serum creatinine > 1.5 mg/dL check units) or hepatic dysfunction (aspartate and alanine transaminase > 50% above normal level), cerebrovascular and central nervous system diseases, history of drug or alcohol abuse, morbid obesity and hearing disorders. No sedative or opioid drugs were administered before the induction of anesthesia. All surgeries were performed under propofol–fentanyl TIVA. The target-controlled infusion (TCI) system Diprifusor® (Astra Zeneca, UK) was used to administer propofol. In the operating room, intravenous and arterial lines were inserted under local anesthesia, and standard monitors were applied (ECG, SpO2). Hemodynamic measurements were carried out with a FloTrac/Vigileo TM System (Edwards, USA). The system consists of a sensor unit (FloTrac) and a stand-alone monitor (Vigileo). It is a pulse wave analysis technique allowing continuous cardiac output measurement. This system uses the arterial pressure waveform to measure the CO detected through a proprietary transducer (FloTrac) attached to a standard arterial line connected to the Vigileo monitor [28, 29] After the surgery, the patients were mechanically ventilated in the intensive care unit (ICU) until full recovery. Simultaneously propofol infusion was maintained until extubation. Pancuronium 0.1 mg/kg was injected to facilitate intubation and then administered as required. This study was initiated with a bolus injection of fentanyl (1.5 μg/kg). The propofol infusion started 5 min later and was maintained with intermittent injections of fentanyl (2–3 μg/kg) administered whenever inadequate analgesia was assessed throughout the surgery, ie. whenever episodes of tachycardia/hypertension in response to surgical stimuli were noted. The bispectral index (BIS; A-2000, Aspect Medical System, Newton, MA) was used to measure the depth of anesthesia and propofol dosage was adjusted to maintain the BIS level between 40 and 60. The BIS uses highly processed electroencephalographic (EEG) signals, acquired from a single self-adhesive forehead sensor, to measure the depth of sedation and hypnosis which is expressed on a unitless scale ranging from 0 to 100 (0, coma or absence of brain electrical activity; 0–40, deep hypnotic state; 40–60, general anesthesia; 60–90, deep to light sedation; and 90–100, awake). The BIS is a complex parameter composed of a combination of time domain, frequency domain and high order spectral subparameters. It is a unique quantitative electroencephalogram parameter (QEEG) which integrates several disparate descriptors of the EEG into a single variable based on a large volume of clinical data, to synthesize a combination that correlates behavioral assessments of sedation and hypnosis yet insensitive to the specific anesthetic agents chosen [2]. During the surgery, crystalloid and colloid fluids were infused according to the following protocol: continuous infusion of crystalloid at a rate of [10 × body weight (kg)] ml/h, interventional colloid infusion to preserve normovolemia (stroke volume variation (SVV) < 12) compensatory to the volume of blood loss. Arterial blood samples (3.5 mL) for plasma propofol and fentanyl concentration measurements were drawn before propofol infusion i.e. 1, 3, 5, 10, 15, 30 min after the beginning of the infusion, then every 30 min until the end of anesthesia and also after 1, 3, 5, 10, 15, 30, 60 min after the termination of propofol infusion for Study 1 and 1, 3, 5, 10, 15, 30, 60 90, 120, 240 min after the termination of propofol infusion for Study 2. The blood samples were transferred into heparinized tubes and centrifuged immediately after collection. Plasma was divided into two equal volumes. Half was stored at 4 °C (propofol analysis) [30] and another half in − 70 °C (fentanyl analysis). The BIS values as well as hemodynamic parameters were recorded continuously throughout the study.

### Analytical method

The propofol concentration in the plasma was measured within 8 weeks by means of high-performance liquid chromatography with fluorescence detection [30]. The limit of quantification was estimated at 10 ng/ml. The within-day coefficients of variation were less than 10%. The fentanyl samples were measured by a validated high-pressure liquid chromatography (Waters 2695 Separation Module, Milford, USA) coupled with a triple quadrupole mass spectrometer, equipped with an electrospray ionization source (ESI+) (Waters Quattro Micro, Milford, USA). The mass spectrometer operated in the multiple-ion monitoring (MRM) mode. Fentanyl and internal standard (IS) were monitored by means of the fragment ions at 387.1→ 238.0 and 532.0→ 219.1, respectively. The column used was a Thermo BDS Hypersil C18 100 × 2.1 mm 3 μm (Thermo Scientific, Waltham, USA). The mobile phase was: formate buffer pH4.0 [A] and acetonitrile [B] (J.T. Baker, Avantor, the Netherlands). The flow rate was 0.2 ml/min, isocratic separation was applied – the mobile phase was used as follows: 70% [B] and 30% [A]. Fentanyl and terconazole (IS) were extracted using a single-step liquid–liquid extraction (LLE) with a mixture of ethyl acetate and hexane. The lower limit of quantification was 0.05 ng/ml for fentanyl using a 0.250 ml sample volume, with a bias of 4.6% and RSD of 5.4%. The calibration curves were linear (r2 ≥ 0.990) over the working range of 0.05–50.0 ng/ml, using 1/× 2 as a weighting factor. Quality control samples at three concentration levels (LQC 0.2 ng/ml, RSD = 9.9%; MQC 1.50 ng/ ml, RSD = 9.7%; HQC 15.0 ng/ml, RSD = 9.4%) were used for validation purposes of the analytical run.

### Model

The population nonlinear mixed-effect modelling was done using NONMEM® (version 7.4 ICON Development, Ellicott City, MD, USA)) and the gfortran compiler. NONMEM runs were executed using Wings for NONMEM (WFN743, https://wfn.sourceforge.net). The FOCE estimation method with the interaction option in NONMEM was applied. The minimum value of the NONMEM objective function (OFV), typical goodness of fit diagnostic plots, and evaluation of the precision of the PK/PD parameter and variability estimates were used to discriminate between various models during the model-building process. The NONMEM data processing, simulations, and plots were carried out using Matlab® Software version 7.0 (The MathWorks, Inc., Natick, MA, USA). The model predictive performance was assessed by means of Visual Predictive Checks (VPC). The VPC calculation was based on 1000 datasets simulated with the final parameter estimates. Different dosing regimens and variable infusion length required the use of prediction corrected VPC (pcVPC) [31]. The pcVPCs were created by correcting the observed and simulated values for the average population prediction in the time-bin divided by population predictions for each observed and simulated value. In this study the 10th, 50th and 90th percentile were used to summarize the data and VPC prediction. The pcVPC enables a comparison of the confidence intervals obtained from prediction with the observed data over time. If the corresponding percentile from the observed data falls outside the 95% confidence interval derived from predictions, it indicates the model misspecification. Since the PK/PD data deviated from nominal times to some extent, binning across time was used. A nonparametric bootstrap was performed to evaluate the uncertainty of final model parameters. Individual patients were randomly sampled with replacement from original dataset to form 300 new data sets with the same number of patients as original dataset. Each new dataset was fitted to the final model and all model parameters were estimated. The bootstrap empirical parameter distributions were summarized as a median with 90% (5th-95th percentile) confidence intervals.

**Fig. 1:**
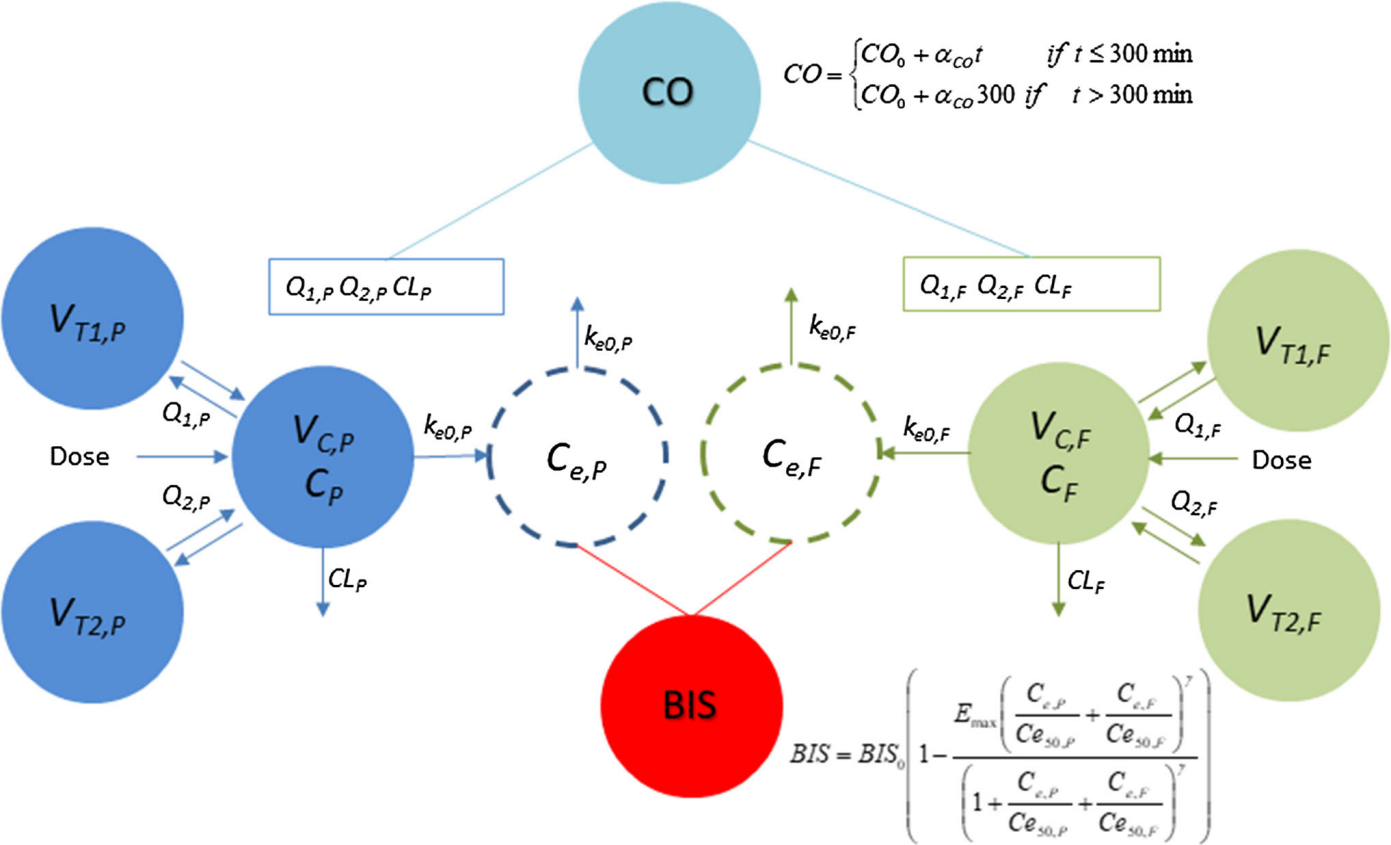
The proposed PK/PD model of propofol, fentanyl, CO and BIS

A schematic representation of the proposed PK/PD model is given in Fig. 1. A three-compartment model was used to describe the PK of both propofol and fentanyl. It was parametrized using systemic clearance, distribution clearance and volumes of distributions. The delay of the anaesthetic effect, with respect to plasma concentrations, was described by an effect compartment. The bispectral index (BIS) was linked to the propofol and fentanyl effect-site concentrations (*C*_*e,P*_ and *C*_*e,F*_) through the following *E*_*max*_ model [32–35]:

1 $$BIS = BIS_{0} \left( {1 - \frac{{E_{\max } \left( {\frac{{C_{e,P} }}{{Ce_{50,P} }} + \frac{{C_{e,F} }}{{Ce_{50,F} }} + \alpha \frac{{C_{e,P} }}{{Ce_{50,P} }}\frac{{C_{e,F} }}{{Ce_{50,F} }}} \right)}}{{\left( {1 + \frac{{C_{e,P} }}{{Ce_{50,P} }} + \frac{{C_{e,F} }}{{Ce_{50,F} }} + \alpha \frac{{C_{e,P} }}{{Ce_{50,P} }}\frac{{C_{e,F} }}{{Ce_{50,F} }}} \right)^{\gamma } }}^{\gamma } } \right)$$

where *C*_*e50,P*_ and *C*_*e50,F*_ denote the concentrations of propofol or fentanyl in the biophase compartment that produce half-maximal decrease in the BIS response, *BIS*_*0*_ denotes the baseline BIS score (fully awake), *E*_*max*_ is the maximal effect fixed to 1 in this work (BIS value of zero at sufficiently high concentrations of propofol or fentanyl), γ is a Hill coefficient also fixed to 1, and α is a first order interaction term (α = 0 suggests additivity, α ≠ 0 suggests nonadditivity). The additive and nonadditve model for drug interactions was explored during the model building process resulting in an estimate of the α parameter not significantly different from 0, suggesting that the interaction between propofol and fentanyl beyond additivity was not supported by the data.

CO was fitted to an empirical linear equation based on the visual inspection of the data:

2 $$CO(t) = \left\{ {\begin{array}{ll} CO_{0} + \alpha_{CO} t & if\;t \le 300\min \\ CO_{0} + \alpha_{CO} 300 & if\,t > 300\min \\ \end{array} } \right.$$

where *CO*_*0*_ is a baseline CO and $\alpha_{CO}$ is a linear rate of change of CO during the surgery. Due to the lack of data, the model assumed that CO was constant after 300 min. This is a very crude assumption and the model should not be extrapolated beyond 300 min, as CO likely returned to the baseline values.

The CO was a priori assumed to affect the distribution and elimination clearances of both drugs. A proportional relationship was assumed (here presented for propofol clearance only):

3 $$CL_{P,i} (t) = \theta_{CLp} \frac{CO(t)}{{6.5}}\exp (\eta_{CLP} )$$

Inter-individual variability (IIV) for all PK/PD parameters was modelled assuming log-normal distribution:

4 $$P_{i} = \theta_{P} \,\text{exp}(\eta_{P,i})$$

where *P*_*i*_ is the set of PK/PD parameters for i_th_ individual, *θ*_*P*_ is the population estimate of PK/PD parameters, *η*_*P,i*_ is a random effect for a particular parameter with mean 0 and variance ω_P_^2^.

Further, any *j*^*th*^ observation of propofol and fentanyl concentration, BIS and CO values for the *i*^*th*^ individual, *C*_*P,obs,ij*_, *C*_*F,obs,ij*_, *BIS*_*obs,ij*_, and *CO*_*obs,ij*_ measured at time *t*_*j*_, were defined by the following equations:

5 $$\begin{aligned} {\text{C}}_{{\text{P,obs,ij }}} &= {\text{ C}}_{{\text{P}}} {(}P_{i} , t_{j} )(1 + \varepsilon_{P,ij} ) \\ {\text{C}}_{{\text{F,obs,ij }}} &= {\text{ C}}_{{\text{F}}} {(}P_{i} , t_{j} )(1 + \varepsilon_{F,ij} ) \\ BIS_{{\text{obs,ij }}} &= BIS(P_{i} , t_{j} ) + \varepsilon_{BIS,ij} \\ CO_{{\text{obs,ij }}} &= CO(P_{i} , t_{j} ) + \varepsilon_{CO,ij} \end{aligned}$$

where *C*_*p*_, *C*_*F*_, *BIS* and *CO* denote the basic structural population model. *P*_i_ are pharmacokinetic parameters for the *i*^*th*^ individual, and *ε*_*Pij*_, *ε*_*Fij*_, *ε*_*BIS,ij*_, *ε*_*CO,ij*_ represent the proportional or additive residual intra-individual random error. We assumed that ε was symmetrically distributed around a mean of 0, with variance denoted by σ^2^.

#### Covariate analysis

Initially, the base model described above was compared to the model with no relationship between CO and PK parameters. For comparison, also the observed values of CO were regressed with PK parameters. Under this scenario, the missing covariates for an individual were obtained by carrying forward the last measured value. Further the classical covariate search was performed by plotting individual (post-hoc) estimates of the PK/PD parameters against covariates (weight, age) to identify their potential effects. Categorical covariates (i.e. study type) were included into the model based on indicator variables. The covariates were added based on biological plausibility and clinical relevance. Also the statistical significance was calculated based on the difference in the minimum of the NONMEM OFV obtained for the two hierarchical models (likelihood ratio). This statistic is approximately χ2 distributed and when the difference in OFV between two nested models is estimated near to 3.84 for one degree of freedom, it corresponds to *p* < 0.05.

#### Model simulations

The final PK/PD model with estimated fixed- and random-effect parameters was used to simulate the concentrations of propofol and fentanyl, the BIS index and CO for an exemplary dosing schemes and patients. Context-sensitive detrimental-time (CSDT) was used to assess the influence of propofol and fentanyl administration on the time required for a decline in the effect compartment concentration upon infusion cessation in relation to patients’ age [36]. The CSDT is the time necessary for a certain decline in virtual effect-site concentration (and consequently increase in BIS) after termination of a continuous infusion of a given duration. This virtual effect-site concentration represents the sum of normalized effect-site concentrations of propofol and fentanyl, assuming an additive interaction between the drugs (*C*_*P,e*_*/Ce*_*50,P*_ + *C*_*F,e*_*/C*_*e50,F*_). The context-sensitive decrement times were simulated based on typical parameter estimates of the final PK/PD model assuming dosing scheme that leads to constant concentrations of propofol and fentanyl (equivalently certain BIS values).

## Results

**Fig. 2:**
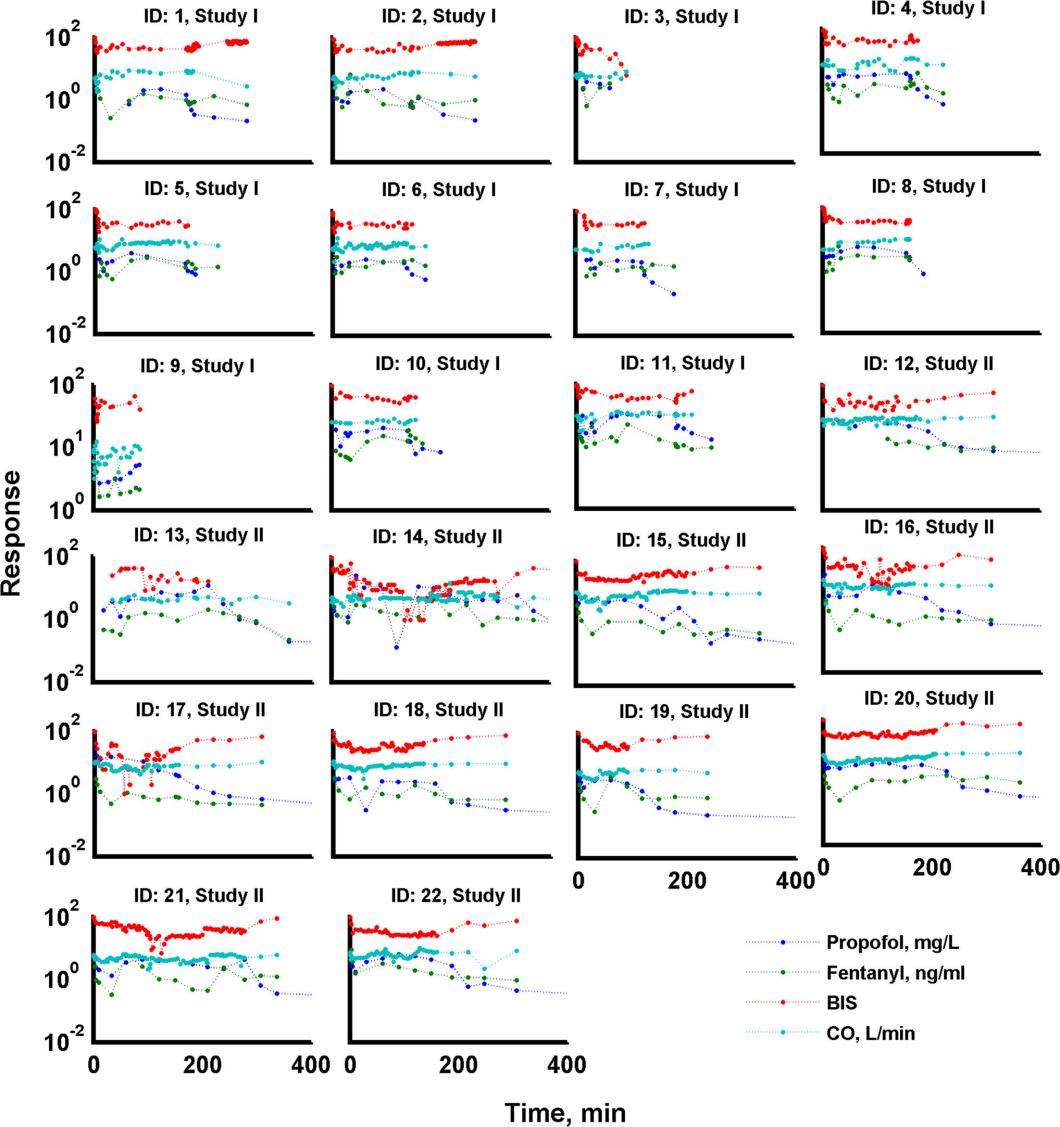
The individual propofol PK, fentanyl PK, BIS and CO time profiles. The dots represent raw measurements. They are connected with straight lines

The data were collected from 22 patients with the demographic characteristic presented in the Table 1 and Tables 1S. The raw observations are presented in Fig. 2.

**Table 1:** Demographic characterization of patients. Results are expressed as median and range

Parameter, unit	Median [range] n = 22
Age, years	64 [51–80]
Height, cm	170 [164–185]
Weight, kg	70 [57.5–105]
BSA, m^2^	1.85 [1.62–2.27]
BMI	24.8 [18.1–35.9]
CI, L/min/m^2^	3.8 [2.5–4.6]
CO, L/min	8.2 [5.0–11]
SYST, mmHg	139 [101–177]
DIAS, mmHg	68.1 [38.4–88.5]
HR, beats/min	75.4 [52.2–94.9]

### PK/PD model

In agreement with the literature the disposition of propofol and fentanyl were described by three compartment mamillary model. The CO was assumed to be proportionally related to the distribution and elimination clearances of propofol and fentanyl. It is consistent with the assumption that both drugs are high-extraction drugs with perfusion limited distribution. The effect compartment and the additive model (Eq. 1 with α fixed to 0) was able to describe the pharmacodynamics response (BIS). The propofol-fentanyl interaction beyond additivity was not supported by the data, likely due to the limitations of the experimental design (e.g. lack of sufficient concentration range of both drugs). Different models regarding CO effects were tested during model building process as summarized in Table 2. A substantial improvement in model fit was noted when model predicted CO was included into the PK/PD model (ΔOFV = 83.349, df = 0). The inclusion of observed CO improved model fits slightly when compared to the model without CO effects on propofol and fentanyl PK (ΔOFV = 5.713, df = 0). Thus, there is a slight benefit of using a model with measured CO as a covariate. Further the addition of patients’ age and study number was found to improve the model fits to the data.

**Table 2:** Nonmem objective function (OFV) for key modeling steps

Model	OFV	ΔOFV
Model without CO effects on propofol and fentanyl PK	7198.025	83.349, df = 0
Base model with observed CO as a covariate: *CO*_*obs*_/6.5	7192.312	77.636, df = 0
Base model: CO/6.5	7114.676	0
Covariates: CO/6.5, α_CO_ ~ AGE	7103.829	− 10.847, df = 1
Covariates: CO/6.5, α_CO_ ~ AGE + STUDY **(Final Model)**	7097.754	− 16.922, df = 2

**Fig. 3:**
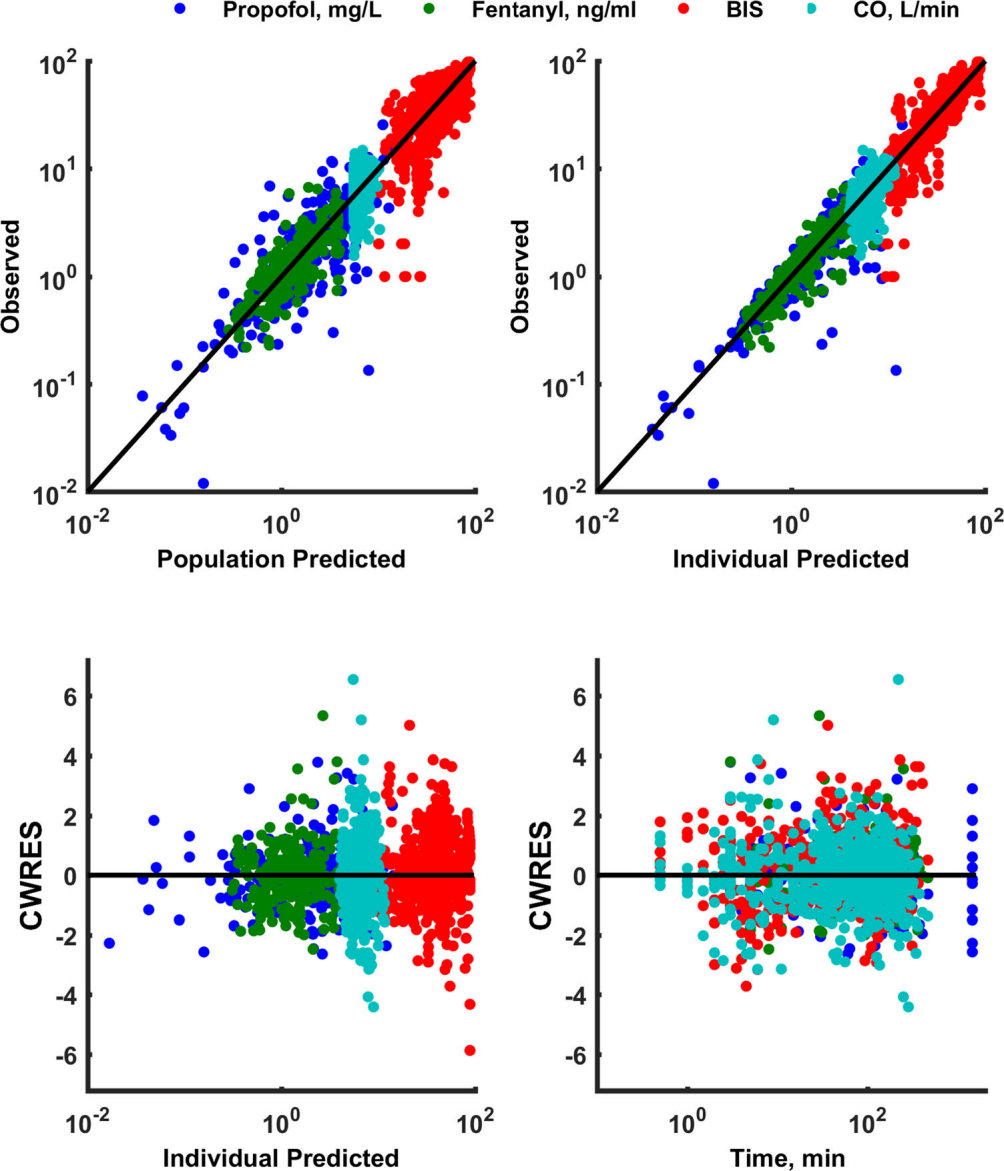
Goodness-of-fit plots for the final PK/PD model: the observed versus the population predicted responses, the observed versus the individual predicted responses, the conditional weighted residuals (CWRES) versus the individual predicted responses, and the CWRES versus time

**Fig. 4:**
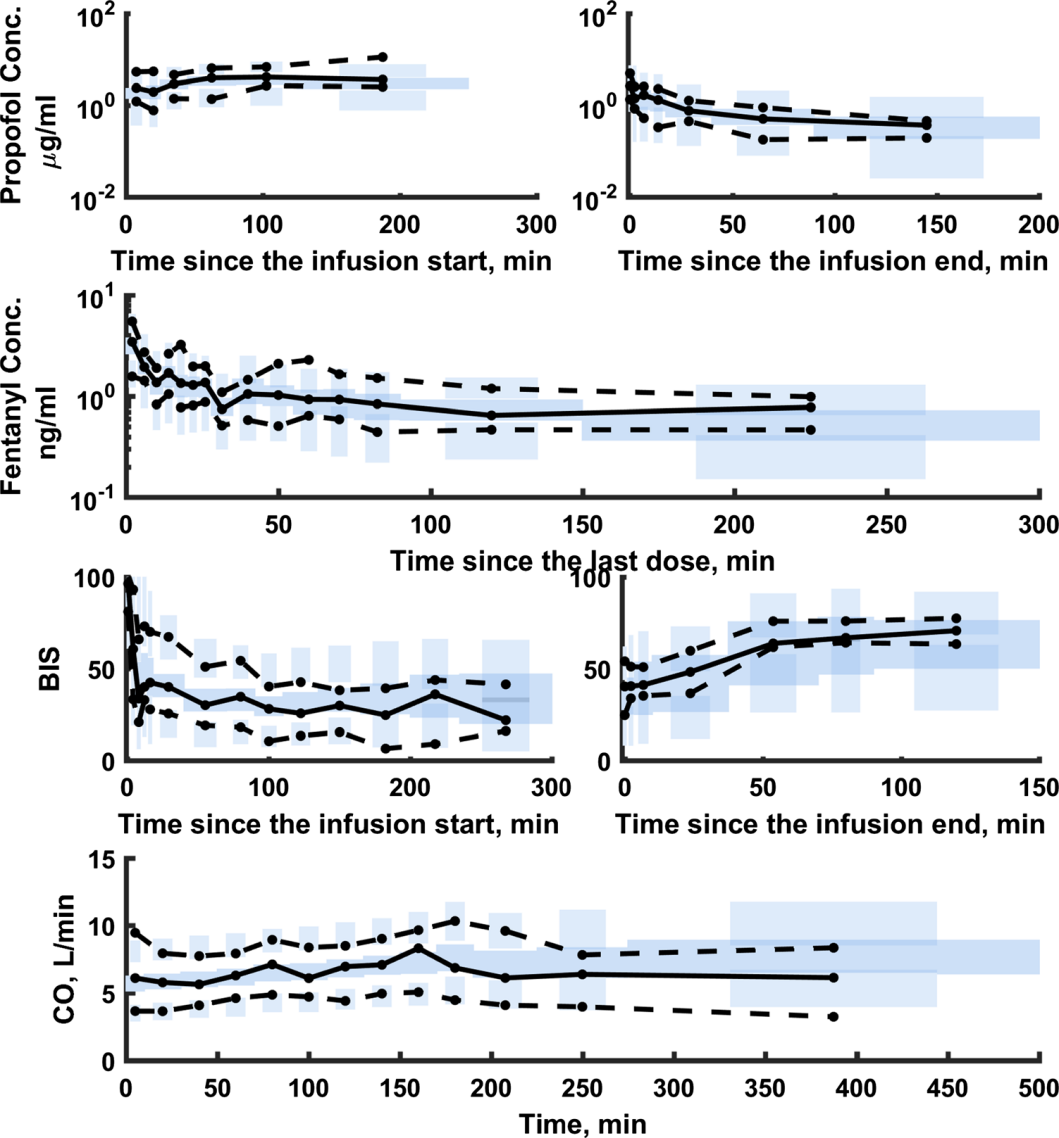
The prediction-corrected VPC plots for the final PK/PD. The VPC plots show the simulation-based 90% confidence intervals around the 10th, 50th, and 90th percentiles of the PK data in the form of blue (50th) and gray (10th and 90th) areas. The corresponding percentiles from the prediction corrected observed data are plotted in black color

The typical goodness-of-fit plots of the final PK/PD model are presented in Fig. 3. The individual predictions for propofol and fentanyl concentrations as well as CO and BIS values were organized around the line of identity. Also weighted residuals showed that the model was reasonably unbiased with respect to the data. The pcVPC plots for the PK/PD measurements are presented in Fig. 4. No major misspecifications were noted for the propofol PK, fentanyl PK, BIS values and CO values indicating agreement between the observation and model prediction. The pcVPC confirms that the model has sufficient predictive performance and can be used to simulate different clinical scenarios if one agrees to all model assumptions. The predicted and measured responses vs. time profiles for each individual are presented in Figs. 1S–4S.

Table 3 shows the final parameter estimates along with the inter- and intra-individual variability. Majority of parameters were estimated with low (lower than 50%) coefficients of variation (CV). The bootstrap confidence intervals show higher uncertainty, especially for between-subject variability parameters. However it can be expected given the overall complexity of the model and a small number of patients included in the study.

**Table 3:** The parameter estimates of the final PK/PD model of propofol and fentanyl. RSE denotes relative standard error of estimate. CI corresponds to bootstrap [5th-95th] confidence interval

Parametr [unit]	θ (RSE,%), CI	%CV (RSE, %) [shrinkage, %], CI
*V*_*C,P*_ [L]	2.42 (40.8), 2.45 [1.43–5.16]	119 (25.3) [29.7], 124 [53.1–192]
*Cl*_*P*_ [L/min]	1.54 (9.2), 1.50 [1.06–1.72]	37.7 (23.4) [1.8], 37.1 [24.1–59.5]
*Q*_*1,P*_ [L/min]	1.89 (24.7), 1.95 [1.40–2.70]	92.1 (24.7) [11.9], 80.2 [38.9–103]
*V*_*T1, P*_ [L]	54.4 (31.6), 53.8 [30.3–88.3]	48.5 (36.0) [27.5], 49.0 [15.8 –87.5]
*Q*_*2,P*_ [L/min]	0.607 (19.6), 0.680 [0.407–1.00]	–
*V*_*T2,P*_ [L]	482 (33.2), 466 [247–3000]	–
*V*_*C,F*_ [L]	25.8 (28.7), 25.8 [16.4–35.9]	37.7 (37.0) [40.7], 38.6 [0.4–72.5]
*CL*_*F*_ [L/min]	0.569 (45.7), 0.555 [0.078–0.917]	52.2 (35.2) [14.9], 52.6 [32.7–146]
*Q*_*1,F*_ [L/min]	5.78 (16.3), 5.7 [4.23–8.18]	54.3 (68.5) [21.6], 44.7 [0.5–104]
*V*_*T1,F*_ [L]	98.9 (16.6), 93 [68.8–139]	42.0 (42.6) [17.5], 39.0 [0.4–75.2]
*Q*_*2,F*_ [L/min]	1.48 (17.4), 1.50 [1.10–1.91]	–
*V*_*T2,F*_ [L]	478 (46.2), 491 [274–939]	–
*BIS*_*0*_ []	88.6 (3.6), 89.0 [85.2–93.4]	–
*C*_*e50,P*_ [mg/L]	2.25 (12.0), 2.34 [1.83–2.86]	46.9 (26.8) [6.6], 47.9 [26.7–66.2]
*C*_*e50,F*_ [ng/ml]	8.77 (124), 8.40 [3.90–26.8]	122 (47) [26.5], 114 [44.2–181]
k_e0P_ = k_e0F_ [L/min]	0.105 (57.1), 0.109 [0.068–0.188]	134 (29.4) [4.8], 129 [76.4–192]
C0_0_, L/min	5.59 (4.5), 5.62 [5.26–5.98]	19.2 (12.0) [3.0], 18.6 [ 15.0–22.5]
α_CO_, (L/min)/h	1.09 (15.2), 1.06 [0.703–1.40]	42.8 (23.7) [13.3], 38.0 [16.1–66.9]
*β*_*AGE*_, %/year	− 3.23 (24.2), − 3.38 [–4.75–( –1.33)]	–
*β*_*STUDY,*_ %	− 50.9 (27.5), − 44.6 [–66.8–(− 7.62)]	–
σ^2^_P,_ %CV	38.6 (10.6), 38.4 [31.2–44.1]	–
σ^2^_F_, %CV	31.0 (9.3), 31.1 [26.0–35.9]	–
σ_BIS_,	8.42 (6.7), 8.36 [7.33–9.38]	–
σ_CO_, L/min	1.41 (6.0), 1.42 [1.28–1.54]	–

**Fig. 5:**
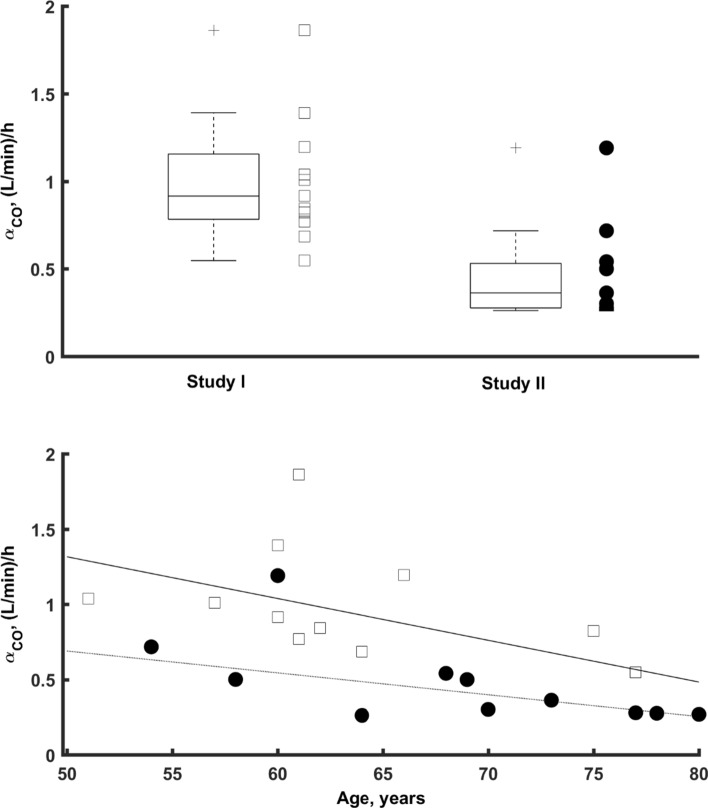
Relationship between the rate of CO output changes *versus* age and study number for all patients included in the study

Figure 5 presents the relationship between the CO rate of change during the surgery and two covariates included into the final model: study and patients’ age. There was a significant and consistent decrease in the rate of change of CO during the surgery ($\alpha_{CO}$) with patients’ age estimated at about 3.23% per year of age. The difference in the observed CO values between two studies was also noted. Patients in Study II had CO rate of change decreased by about 50.9% in comparison to patients in Study I.

### Simulations

**Fig. 6:**
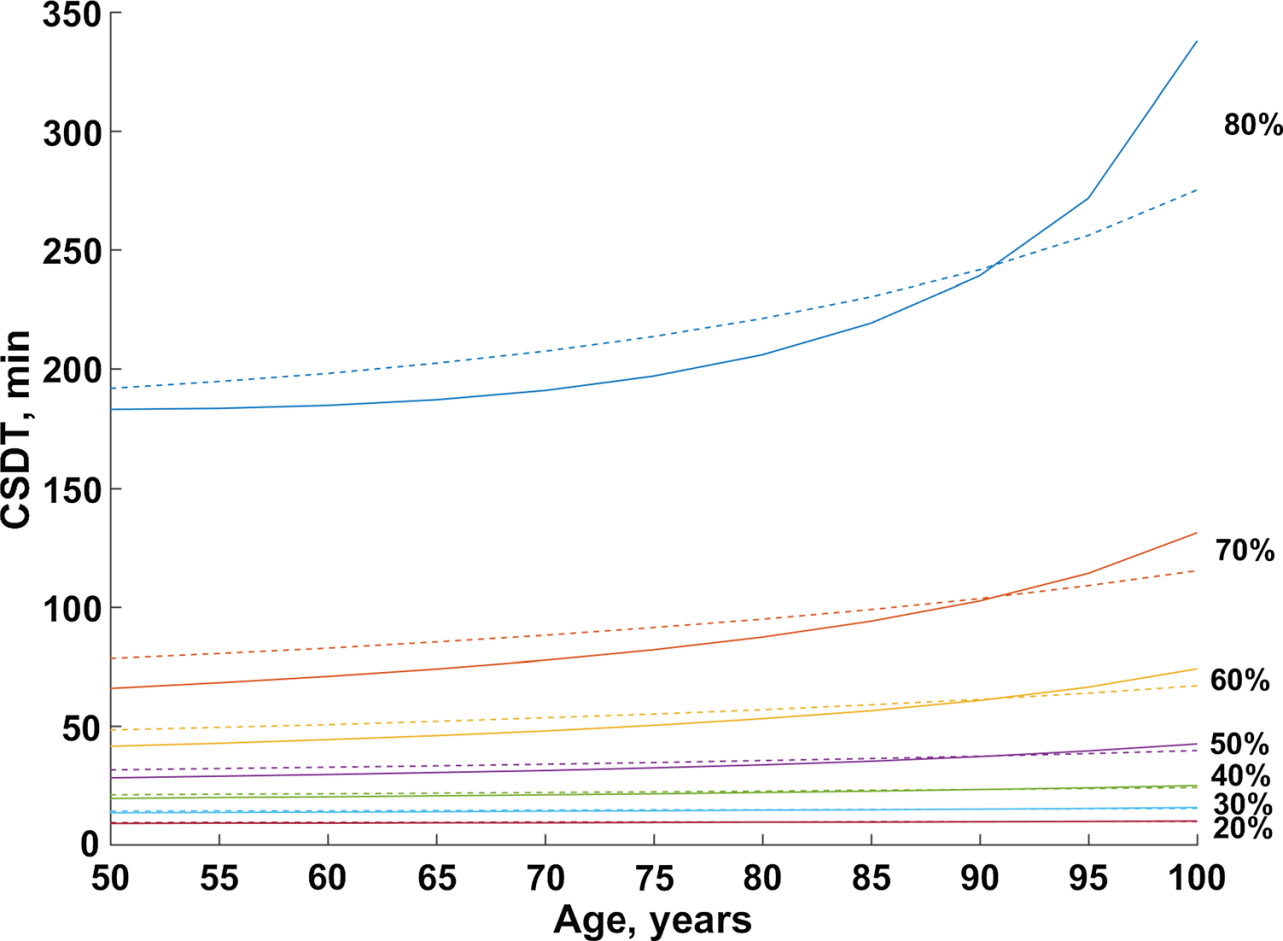
Context-sensitive effect-site decrement times (CSDT) for propofol-fentanyl infusions showing the time for decreasing the effect-site concentrations of a given percentage (20–80%) from the maintained effect-site concentration after propofol/fentanyl infusion cessations for subjects of different age. The solid and dashed lines corresponds to study I and study II predictions. The propofol and fentanyl biophase concentrations were kept at 3.0 mg/l and 1.5 ng/ml respectively for 200 min, which corresponds to the BIS values of about 35. CSDT of 80%, 70%, …, 20% corresponds to achieving a BIS values of 77, 68, 61, 55, 50, 46, 43

Figure 6 presents the dependence of the CSDT on patients’ age for a propofol-fentanyl infusion that led to propofol and fentanyl biophase concentrations of 3.0 mg/l and 1.5 ng/ml for 200 min. These concentrations correspond to the BIS values of about 35**.** The CSDT corresponds to time needed to decrease the effect-site concertation (and consequently increase BIS) by a given percent. The CSDT increases with age of the patients. Also some between-study difference was noted. As an example, BIS value of 61 (corresponding to 60% in Fig. 6) was achieved in 41 min (Study I) and 48 min (Study II) for a 50 years of age patient, and 53 min (Study I) and 57 min (Study II) for a 80 years old patient after infusion cessation that was kept at BIS value of 35 for 200 min. The time required to achieve 80% decrease was 129 min (Study 1) or 183 min (Study 2) for a 50 years of age patient and 206 min (Study 1) and 221 min (Study 2) for a 80 years of age patient.

Figure 5S presents the simulation using the final model of propofol and fentanyl concentrations i.e. BIS values and CO values after intravenous infusion of both drugs in relation to 50, 65 and 80 years old patient (patients are shown for Study II) for the artificial dosing protocol. The patients’ age related differences can be observed in this plot. The CO increased most rapidly for the youngest subjects. This difference influences the recovery from anesthesia. Basically, the higher CO values in younger patients, the lower the propofol and fentanyl concentrations and higher the BIS values, especially at the end of surgery. Figure 6S shows the influence of different fentanyl doses on the responses measured in a 65-year-old patient. For the same propofol dosing scheme, the BIS values showed small dose-dependent variations around each fentanyl dose. Also different rates of BIS increase after infusion cessation were predicted.

## Discussion

To our best knowledge, this is the first study assessing the influence of CO on the PK/PD of propofol and fentanyl during TIVA in patients undergoing abdominal aortic surgery. To assess the usefulness of CO measurement for propofol and fentanyl dosing, we proposed a PK/PD model that took blood flow into account and used BIS as an efficacy response.

### Model structure and parameters

The influence of fentanyl on the recovery from propofol-fentanyl TIVA has already been demonstrated by our group [23] however without the inclusion of CO effects. In the present study, a three compartment model was used to describe the concentration–time profiles of both propofol and fentanyl. It clearly removes bias in model parameters introduced by using a two compartment model. As an example, the elimination clearance of propofol was estimated at 1.54 L/min, whereas in our earlier studies at 2.64 L/min and 2.22 L/min [23, 38]. Eleveld et al. in study based on 21 previously published data sets [39] used a three-compartment PK model for propofol PK with the elimination clearance estimated at 1.53 L/min, which is very close to our typical value of CL (1.54 L/min). Considering the distribution of propofol, a membrane barrier has been postulated to separate the slow distribution compartment [13], assuming flow dependent distribution to the well-perfused tissues and membrane permeability limited distribution to the second tissue compartment. In our study, for propofol the sum of peripheral compartment clearence (*Q*_*1,P*_ + *Q*_*2,P*_) was smaller compared to fentanyl (2.50 L/min vs 7.26 L/min) and smaller than the CO value (6.5 L/min) therefore some contribution of permeability limited kinetics (not dependent on CO) might be expected for propofol.

The typical values of *C*_*e50*_ of propofol (2.25 mg/L) and fentanyl (8.77 ng/ml) are consistent with the literature data regarding PD of both drugs with the narcotic EEG effect as a PD response. For propofol *C*_*e50*_, for BIS as a PD effect was estimated at 2.71–3.44 mg /L [40], whereas for fentanyl the *C*_*e50*_ with EEG power spectrum analysis is equal to 7–10 ng/ml [41].

### The influence of patients' age

The patients’ age was shown to be a significant covariate associated with the rate of change of CO during the surgery. The same phenomenon was noted by Heilbrunn and Allbritten [37] who examined the cardiac output changes during and after surgical procedures. However, in our study, the time-related elevation in CO was smaller for older patients. This can be explained by the decrease in the cardiac functional reserve occurring as an effect of aging [42, 43]. In our study, CO changes directly influenced propofol and fentanyl disposition and thus also affected the BIS values. However, due to the age-related blunting of the CO increase at the end of the surgery, older patients achieved higher propofol and fentanyl concentrations resulting in lower BIS values (Figs. 5 and 5s) which further affected the recovery profile. This is in agreement with the theoretical paradigm related to age related changes in PK, which points to the diminished liver blood flow being responsible for the slower elimination rate of highly extracted drugs [44, 45]. However, this effect was not large within the range of infusion durations under study. As presented in Fig. 6, the difference between 50 and 80 years of age patients in the 60% and 80% decrement times of propofol-fentanyl infusion is estimated at about 10 or 25 min, respectively. This effect could be much more significant for infusions lasting more than 4 h, due to fentanyl accumulation. However, due to a small impact of fentanyl on BIS values when compared to propofol, the differences in recovery of consciousness would be less visible [46]. Another issue is the recovery of spontaneous breathing which is known to be affected by opioids. Unfortunately, this aspect cannot be assessed based on this data only.

### Model simulations and clinical significance

The clinical consequences of the results of this study are presented in Figs. 5S and 6S which show some examples of simulations performed based on the final PK/PD model. At the age of 50, higher CO values were achieved at the end of surgery when compared to the age of 65 and 80. This resulted in lower propofol and fentanyl concentrations, higher BIS values and as a consequence faster recovery (Fig. 5S). The differences were most visible at the recovery period which is consistent with the physiologically-based, recirculatory model of the kinetics and dynamics of propofol in a man and developed by Upton and Ludbrook [13]. In this model, similar to our result, the changes in CO had only minor effect on the time of loss of consciousness but largely affected the time to recovery.

The proposed PK/PD model might be useful in optimizing the dosing of propofol and fentanyl during TIVA in patients undergoing abdominal aortic surgery. The model suggests that the predicted CO values could be more precise in adjusting the propofol and fentanyl doses than CO values directly measured throughout the surgery. The less significant impact of the measured CO on PK of studied drugs can be related to the fact that (a) the measured CO only approximates organ blood flow and (b) it is a measured variable that is stochastically related to the true value of liver and tissue blood flow of each subject. The inclusion of CO into the model led to a small decrease (less than about 20%) in inter-individual variability of PK parameters as presented in Table 2S. It indicates rather limited clinical applicability of the final model in predicting drug dosing in comparison to a simple model (without CO effect on PK/PD parameters). Please note that in the final model the variability in dose-rate leading to similar steady-state concentration across the subjects is a combination of inter-individual variability in PK and CO parameters. The latter source of variability can be decreased by conditioning propofol and fentanyl dosing decisions on the previously measured CO in an individual subject. In principle, it could lead to more precise dosing for an individual subject. However, based on model simulations, even an exact knowledge of CO changes in an individual subjects, would lead to a decrease in drug-dosing variability of at most 20%. It makes CO measurements of rather limited usefulness in guiding propofol and fentanyl dosing, despite clear casual effect of CO on clearances of both drugs.

The elimination and distribution clearance of highly-extracted and perfusion limited drugs is flow-dependent. The hepato-splanchnic blood flow reaches 25–30% of the CO [47]. However, some changes in the distribution of CO during cardiovascular surgeries cannot be excluded due to the different adaptation processes [22]. Peeters et al. [4] in a pilot study, examined the influence of the CO and liver blood flow on the clearance of propofol in five critically ill patients. They noted that liver blood flow is a more predictive indicator of propofol clearance than measured CO in the studied population. It is in agreement with our study, as the model predicted CO is a better surrogate of liver blood flow than measured CO. Similarly, the animal studies confirm the significance of CO in predicting propofol concentrations during constant infusion [48–50]. In all these studies the plasma remifentanil and propofol concentrations were influenced by CO during continuous infusions, with concentrations decreasing with increased CO and increasing with decreased CO. In our study CO changes occurred as a result of inter- and intra-patient variability connected with patients’ individual characteristics and clinical scenario, whereas in the animal studies by Kurita et al. [48–50], the animals were divided into groups based on the CO maintained throughout the study. Also, it is consistent with the physiologically-based models of propofol or fentanyl [13, 51] which show CO as a main determinant of drug clearances. Another clinical implication of our study is related to the interaction between propofol and fentanyl. The interaction between propofol and opioids was studied extensively in the literature where the differences in propofol hypnosis were observed depending on the type of opioid used [18, 52, 53]. One of the proposed reasons of such differences is the pharmacokinetic hypothesis related to the opioid-driven changes in CO. In our work we were unable to show the effects of either propofol or fentanyl on CO, instead we used an empirical relationship describing the surgery-related increase in CO.

In summary, we illustrated the widely recognized theoretical paradigm of the relationship between CO and the clearance of highly extracted drugs and distribution clearance of perfusion-rate limited drugs, under real clinical scenarios. For that purpose a PK/PD model was built to describe the relationship between propofol and fentanyl dosing, the measured CO and BIS values in patients undergoing abdominal aortic surgery. The uncertainty and the indirectness of the measured CO to liver and tissue blood flow required using a model predicted CO values as a predictor. The measured CO values were shown to be of rather limited usefulness for propofol and fentanyl dose-adjustment in patients undergoing abdominal aortic surgery. The patients‘ age was identified as a covariate for the observed CO changes during anesthesia. Thus, patients’ age can be associated with different PK profiles, depth of anesthesia and recovery profiles.

## Electronic supplementary material

Below is the link to the electronic supplementary material.

Supplementary file1 (DOCX 1774 kb)

## References

[1] Kalkman CJ, Peelen LM, Moons KG (2011). Pick up the pieces depth of anesthesia and long-term mortality. Anesthesiology.

[2] Liu YH, Qiu DJ, Jia L, Tan JT, Kang JM, Xie T, Xu HM (2019). Depth of anesthesia measured by bispectral index and postoperative mortality: a meta-analysis of observational studies. J Clin Anesth.

[3] Hiraoka H, Yamamoto K, Okano N, Morita T, Goto F, Horiuchi R (2004). Changes in drug plasma concentrations of an extensively bound and highly extracted drug, propofol, in response to altered plasma binding. Clin Pharmacol Ther.

[4] Peeters MY, Aarts LP, Boom FA, Bras LJ, Tibboel D, Danhof M, Knibbe CA (2008). Pilot study on the influence of liver blood flow and cardiac output on the clearance of propofol in critically ill patients. Eur J Clin Pharmacol.

[5] Upton RN, Ludbrook GL (1997). A physiological model of induction of anaesthesia with propofol in sheep. 1. Structure and estimation of variables. Br J Anaesth.

[6] Bjornsson MA, Norberg A, Kalman S, Karlsson MO, Simonsson US (2010). A two-compartment effect site model describes the bispectral index after different rates of propofol infusion. J Pharmacokinet Pharmacodyn.

[7] Schuttler J, Ihmsen H (2000). Population pharmacokinetics of propofol: a multicenter study. Anesthesiology.

[8] Shafer SL (1993). Advances in propofol pharmacokinetics and pharmacodynamics. J Clin Anesth.

[9] Bienert A, Kusza K, Wawrzyniak K, Grzeskowiak E, Kokot ZJ, Matysiak J, Grabowski T, Wolc A, Wiczling P, Regulski M (2010). Assessing circadian rhythms in propofol PK and PD during prolonged infusion in ICU patients. J Pharmacokinet Pharmacodyn.

[10] Knibbe CA, Zuideveld KP, DeJongh J, Kuks PF, Aarts LP, Danhof M (2002). Population pharmacokinetic and pharmacodynamic modeling of propofol for long-term sedation in critically ill patients: a comparison between propofol 6% and propofol 1%. Clin Pharmacol Ther.

[11] Peeters MY, Bras LJ, DeJongh J, Wesselink RM, Aarts LP, Danhof M, Knibbe CA (2008). Disease severity is a major determinant for the pharmacodynamics of propofol in critically ill patients. Clin Pharmacol Ther.

[12] Levitt DG, Schnider TW (2005). Human physiologically based pharmacokinetic model for propofol. BMC Anesthesiol.

[13] Upton RN, Ludbrook G (2005). A physiologically based, recirculatory model of the kinetics and dynamics of propofol in man. Anesthesiology.

[14] Upton RN, Ludbrook GL (1999). A model of the kinetics and dynamics of induction of anaesthesia in sheep: variable estimation for thiopental and comparison with propofol. Br J Anaesth.

[15] Marsh B, White M, Morton N, Kenny GN (1991). Pharmacokinetic model driven infusion of propofol in children. Br J Anaesth.

[16] Schnider TW, Minto CF, Gambus PL, Andresen C, Goodale DB, Shafer SL, Youngs EJ (1998). The influence of method of administration and covariates on the pharmacokinetics of propofol in adult volunteers. Anesthesiology.

[17] Bienert A, Zaba Z, Grzeskowiak E, Kusza K, Grabowski T (2009). Pharmacokinetics and pharmacodynamics of propofol during propofol-alfentanil and propofol-remifentanil total intravenous anaesthesia monitored by spectral frequency index. Med Sci Monit.

[18] Mertens MJ, Olofsen E, Burm AG, Bovill JG, Vuyk J (2004). Mixed-effects modeling of the influence of alfentanil on propofol pharmacokinetics. Anesthesiology.

[19] Hug CC, McLeskey CH, Nahrwold ML, Roizen MF, Stanley TH, Thisted RA, Walawander CA, White PF, Apfelbaum JL, Grasela TH (1993). Hemodynamic effects of propofol: data from over 25,000 patients. Anesth Analg.

[20] Upton RN, Ludbrook GL, Grant C, Martinez AM (1999). Cardiac output is a determinant of the initial concentrations of propofol after short-infusion administration. Anesth Analg.

[21] Birkholz T, Leuthold C, Schmidt J, Ihmsen H, Schüttler J, Jeleazcov C (2018). Influence of cardiac output on the pharmacokinetics of sufentanil in anesthetized pigs. Anesthesiology.

[22] Siegemund M, van Bommel J, Stegenga ME, Studer W, van Iterson M, Annaheim S, Mebazaa A, Ince C (2010). Aortic cross-clamping and reperfusion in pigs reduces microvascular oxygenation by altered systemic and regional blood flow distribution. Anesth Analg.

[23] Wiczling P, Bieda K, Przybyłowski K, Hartmann-Sobczyńska R, Borsuk A, Matysiak J, Kokot JZ, Sobczyński P, Grześkowiak E, Bienert A (2016). Pharmacokinetics and pharmacodynamics of propofol and fentanyl in patients undergoing abdominal aortic surgery – a study of pharmacodynamic drug–drug interactions. Biopharmaceutics and Drug.

[24] Jain U (1996). Perioperative use of propofol for cardiac surgery. J Clin Anesth.

[25] Venkataraman R (2006) Vascular surgery critical care. perioperative cardiac optimization to improve survival. Crit Care Med; 34: S200–207. 10.1097/01.ccm.0000231885.74567.4f 10.1097/01.CCM.0000231885.74567.4F 16917424

[26] Shine TS, Murray MJ (2004). Intraoperative management of aortic aneurysm surgery. Anesthesiol Clin North America.

[27] Bjelland TW, Klepstad P, Haugen BO, Nilsen T, Dale O (2013). Effects of hypothermia on the disposition of morphine, midazolam, fentanyl, and propofol in intensive care unit patients. Drug Metab Dispos.

[28] Alhashemi JA, Cecconi M, Hofer CK (2011). Cardiac output monitoring: an integrative perspective. Crit Care.

[29] A. Hendy, Ş. Bubenek. Pulse waveform hemodynamic monitoring devices: recent advances and the place in goal-directed therapy in cardiac surgical patients. Pulse waveform hemodynamic monitoring devices: recent advances and the place in goal-directed therapy in cardiac surgical patients. Rom J Anaesth Intensive Care 23(1): 55–65. 10.21454/rjaic.7518.231.wvf 10.21454/rjaic.7518.231.wvf PMC5505364 28913477

[30] Plummer GF (1987). Improved method for the determination of propofol in blood by high-performance liquid chromatography with fluorescence detection. J Chromatograph B.

[31] Bergstrand M, Hooker AC, Wallin JE, Karlsson MO (2011). Prediction-corrected visual predictive checks for diagnosing nonlinear mixed-effects models AAPS J.

[32] Ben-Shlomo I, Finger J, Barav E, Perl A, Etchin A, Tverskoy M (1993). Propofol and fentanyl act additively for induction of anesthesia. Anaesthesia.

[33] Bouillon T, Bruhn J, Radulescu L, Corina Andresen, Thomas J Shafer, Carol Cohane, Steven L Shafer (2004) Pharmacodynamic interaction between propofol and remifentanil regarding hypnosis, tolerance of laryngoscopy, bispectral index, and electroencephalographic approximate entropy. Anesthesiology 100(6):1353–137 2. 10.1097/00000542-200406000-00006. 10.1097/00000542-200406000-00006 15166553

[34] Hannam JA, Short TG (2019). Drug Interactions: Additivity and Synergy among Anaesthetic Drugs. Personalized Anaesthesia.

[35] Minto C, Schnider T, Short T, Gregg K, Gentilini A, Shafer S (2000). Response surface model for anesthetic drug interactions. Anesthesiology.

[36] Hughes MA, Glass PS, Jacobs JR (1992). Context-sensitive half-time in multicompartment pharmacokinetic models for intravenous anesthetic drugs. Anesthesiology.

[37] Ludbrook GL, Upton RN (2003). Pharmacokinetic drug interaction between propofol and remifentanil?. Anesth Analg.

[38] Wiczling P, Bienert A, Sobczyński P, Hartmann-Sobczyńska R, Bieda K, Marcinkowska A, Malatyńska M, Kaliszan R, Grześkowiak E (2012). Pharmacokinetics and pharmacodynamics of propofol in patients undergoing abdominal aortic surgery. Pharmacol Rep.

[39] Eleveld DJ, Proost JH, Cortínez LI, Absalom AR, Struys MM (2014). A general purpose pharmacokinetic model for propofol. Anesth Analg.

[40] Sahinovic MM, Struys MMRF, Absalom AR (2018). Clinical Pharmacokinetics and Pharmacodynamics of Propofol. Clin Pharmacokinet.

[41] Lotsch J (2005) Updates of the Clinical Pharmacology of Opioids with Special Attention to Long-Acting Drugs. Pharmacokinetic–Pharmacodynamic Modeling of Opioids. Journal of Pain and Symptom Management 29(5S): S90-S102. 10.1016/j.jpainsymman.2005.01.012 10.1016/j.jpainsymman.2005.01.012 15907650

[42] Heilbrunn A, Allbritten FF (1960). Cardiac Output During and Following Surgical Operations Ann Surg.

[43] Xuming Dai, Scott L Hummel, Jorge B Salazar, George E Taffet, Susan Zieman, and Janice B Schwartz (2015) Cardiovascular physiology in the older adults J Geriatr Cardiol 12(3): 196–201. 10.11909/j.issn.1671-5411.2015.03.015 PMC4460159 26089840

[44] Castillo JG, Silvay G, Chikwe J (2009). Cardiac anesthesia and surgery in geriatric patients: epidemiology, current surgical outcomes, and future directions hsr proc intensive carecardiac anesthesia and surgery in geriatric patients: epidemiology. Cardiovasc Anesth.

[45] Cusack BJ (2004). Pharmacokinetics in older person. Am J Geriatr Pharmacother.

[46] Lysakowski C, Dumont L, Pellegrini M, Clergue F, Tassony E (2001). Effects of Fentanyl, Alfentanil, Remifentanil and Sufentanil on Loss of Consciousness and Bispectral Index During Propofol Induction of Anaesthesia. Br J Anaesth.

[47] Dahn MS, Lange P, Lobdell K, Hans B, Jacobs LA, Mitchell RA (1987). Splanchnic and total body oxygen consumption differences in septic and injuried patients. Surgery.

[48] TassonyiKurita T, Uraoka M, Morita K, Suzuki M, Morishima Y, Sato S (2011). Influence of haemorrhage on the pseudo-steady-state remifentanil concentration in a swine model: a comparison with propofol and the effect of haemorrhagic shock stage. Br J Anaesth.

[49] Kurita T, Uraoka M, Jiang Q, Suzuki M, Morishima Y, Morita K, Sato S (2013). Influence of cardiac output on the pseudo-steady state remifentanil and propofol concentrations in swine. Acta Anaesthesiol Scand.

[50] Kurita T, Morita K, Kazama T, Sato S (2002). Influence of cardiac output on plasma propofol concentrations during constant infusion in swine. Anesthesiology.

[51] Upton RN, Foster DJ, Christrup LL, Dale O, Moksnes K, Popper L (2012). A physiologically-based recirculatory meta-model for nasal fentanyl in man. J Pharmacokinet Pharmacodyn.

[52] Bienert A, Wiczling P, Zaba C, Zaba Z, Wolc A, Marciniak R, Grzeskowiak E, Kusza K (2012). Influence of demographic factors, basic blood test parameters and opioid type on propofol pharmacokinetics and pharmacodynamics in ASA I-III patients. Arzneimittelforschung.

[53] Koitabashi T, Johansen JW, Sebel PS (2002). Remifentanil dose/electroencephalogram bispectral response during combined propofol/regional anesthesia. Anesth Analg.

