# Supplementary material for: The influence of cardiac output on propofol and fentanyl pharmacokinetics and pharmacodynamics in patients undergoing abdominal aortic surgery
Source: J Pharmacokinet Pharmacodyn. 2020 Aug 25;47(6):583–96. doi: 10.1007/s10928-020-09712-1 (PMC7652808; doi:10.1007/s10928-020-09712-1)
Supplement: Supplementary file 1 — Supplementary file1 (DOCX 1774 kb) [file 10928_2020_9712_MOESM1_ESM.docx]

**Supplementary Materials for:**

**The influence of cardiac output on propofol and fentanyl pharmacokinetics and**

**pharmacodynamics in patients undergoing abdominal aortic surgery.**

Agnieszka Bienert (1), Paweł Sobczyński (2), Katarzyna Młodawska (1), Roma Hartmann-Sobczyńska (3), Edmund Grześkowiak (1), Paweł Wiczling (4)

**Supplementary Materials:**

Table 1S. Demographic data for individual patients.

| **ID** | **Study** | **Age**  **years** | **Height**  **cm** | **Weight**  **kg** | **BMI** | **BSA**  **m^2^** | **Average CI**  **L/min/m^2^** | **Average CO**  **L/min** | **Average SBP**  **mmHg** | **Average DBP**  **mmHg** | **Average HR**  **beats/min** |
| --- | --- | --- | --- | --- | --- | --- | --- | --- | --- | --- | --- |
| 1 | I | 61 | 185 | 100 | 29.2 | 2.27 | 3.3 | 7.2 | 131 | 67.7 | 66.1 |
| 2 | I | 75 | 167 | 60 | 21.5 | 1.67 | 3.7 | 6.6 | 159 | 66.3 | 67.9 |
| 3 | I | 77 | 170 | 72 | 24.9 | 1.84 | 3.9 | 7.0 | 127 | 71.4 | 95.0 |
| 4 | I | 57 | 168 | 82.5 | 28.6 | 1.96 | 3.7 | 8.8 | 137 | 61.4 | 59.6 |
| 5 | I | 60 | 170 | 64 | 22.2 | 1.74 | 4.5 | 8.8 | 154 | 80.6 | 73.9 |
| 6 | I | 62 | 170 | 62 | 21.4 | 1.71 | 4.4 | 8.6 | 153 | 80.1 | 92.0 |
| 7 | I | 60 | 168 | 66 | 22.8 | 1.76 | 4.6 | 9.0 | 152 | 88.5 | 82.5 |
| 8 | I | 61 | 183 | 72.5 | 25.1 | 1.92 | 4.5 | 9.8 | 160 | 69.5 | 62.1 |
| 9 | I | 66 | 169 | 85 | 29.4 | 2.00 | 4.4 | 9.9 | 177 | 74.8 | 72.4 |
| 10 | I | 64 | 164 | 60 | 20.8 | 1.65 | 4.2 | 7.5 | 164 | 70.3 | 84.2 |
| 11 | I | 51 | 175 | 96 | 33.2 | 2.16 | 4.6 | 10.5 | 141 | 88.3 | 87.5 |
| 12 | II | 69 | 172 | 102 | 34.5 | 2.21 | 3.6 | 9.0 | 160 | 76.6 | 54.1 |
| 13 | II | 64 | 165 | 57.5 | 21.1 | 1.62 | 2.7 | 5.0 | 109 | 61.9 | 76.8 |
| 14 | II | 73 | 165 | 67 | 24.6 | 1.75 | 2.8 | 5.8 | 118 | 68.6 | 90.0 |
| 15 | II | 60 | 170 | 93 | 32.2 | 2.10 | 4.0 | 11 | 130 | 67.7 | 71.3 |
| 16 | II | 77 | 175 | 79 | 25.8 | 1.96 | 3.0 | 6.4 | 101 | 52.9 | 76.8 |
| 17 | II | 70 | 171 | 105 | 35.9 | 2.23 | 3.5 | 8.3 | 130 | 38.4 | 52.2 |
| 18 | II | 68 | 168 | 68 | 24.1 | 1.78 | 4.6 | 9.2 | 133 | 66.8 | 84.9 |
| 19 | II | 78 | 185 | 67 | 19.6 | 1.86 | 3.0 | 6.2 | 148 | 64.0 | 64.7 |
| 20 | II | 54 | 164 | 58.5 | 21.8 | 1.63 | 4.0 | 7.6 | 131 | 66.7 | 86.3 |
| 21 | II | 80 | 178 | 80 | 25.2 | 1.99 | 2.5 | 5.5 | 112 | 59.5 | 62.4 |
| 22 | II | 58 | 179 | 58 | 18.1 | 1.70 | 3.7 | 8.0 | 142 | 76.1 | 84.9 |

Table 2S. Difference in inter-individual variability of PK/PD parameters between the model without CO effects on propofol and fentanyl PK (initial model) and the final model.

| **Parametr [unit]** | **%CV Initial model** | **%CV Final Model** | **Difference, %** |
| --- | --- | --- | --- |
| *V_C,P_* [L] | 113 | 119 | 6.0 |
| *Cl_P_* [L/min] | 33.3 | 37.7 | 4.4 |
| *Q_1,P_* [L/min] | 90.3 | 92.1 | – 1.8 |
| *V_T1, P_* [L] | 44.9 | 48.5 | – 3.6 |
| *Q_2,P_* [L/min] | - | - | - |
| *V_T2,P_* [L] | - | - | - |
| *V_C,F_* [L] | 38.7 | 37.7 | – 1.0 |
| *CL_F_* [L/min] | 59.3 | 52.2 | – 7.1 |
| *Q_1,F_* [L/min] | 57.0 | 54.3 | – 2.7 |
| *V_T1,F_* [L] | 47.9 | 42.0 | – 5.9 |
| *Q_2,F_* [L/min] | - | - | - |
| *V_T2,F_* [L] | - | - | - |
| *BIS_0_* [] | - | - | - |
| *C_e50,P_* [mg/L] | 49.2 | 46.9 | 0.7 |
| *C_e50,F_* [ng/ml] | 119 | 122 | 3.0 |
| k_e0P_ = k_e0F_ [L/min] | 133 | 134 | 1.0 |
| C0_0_, L/min | 19.2 | 19.2 | 0.0 |
| α_CO_, (L/min)/h | 67.6 | 42.8 | – 24.8 |


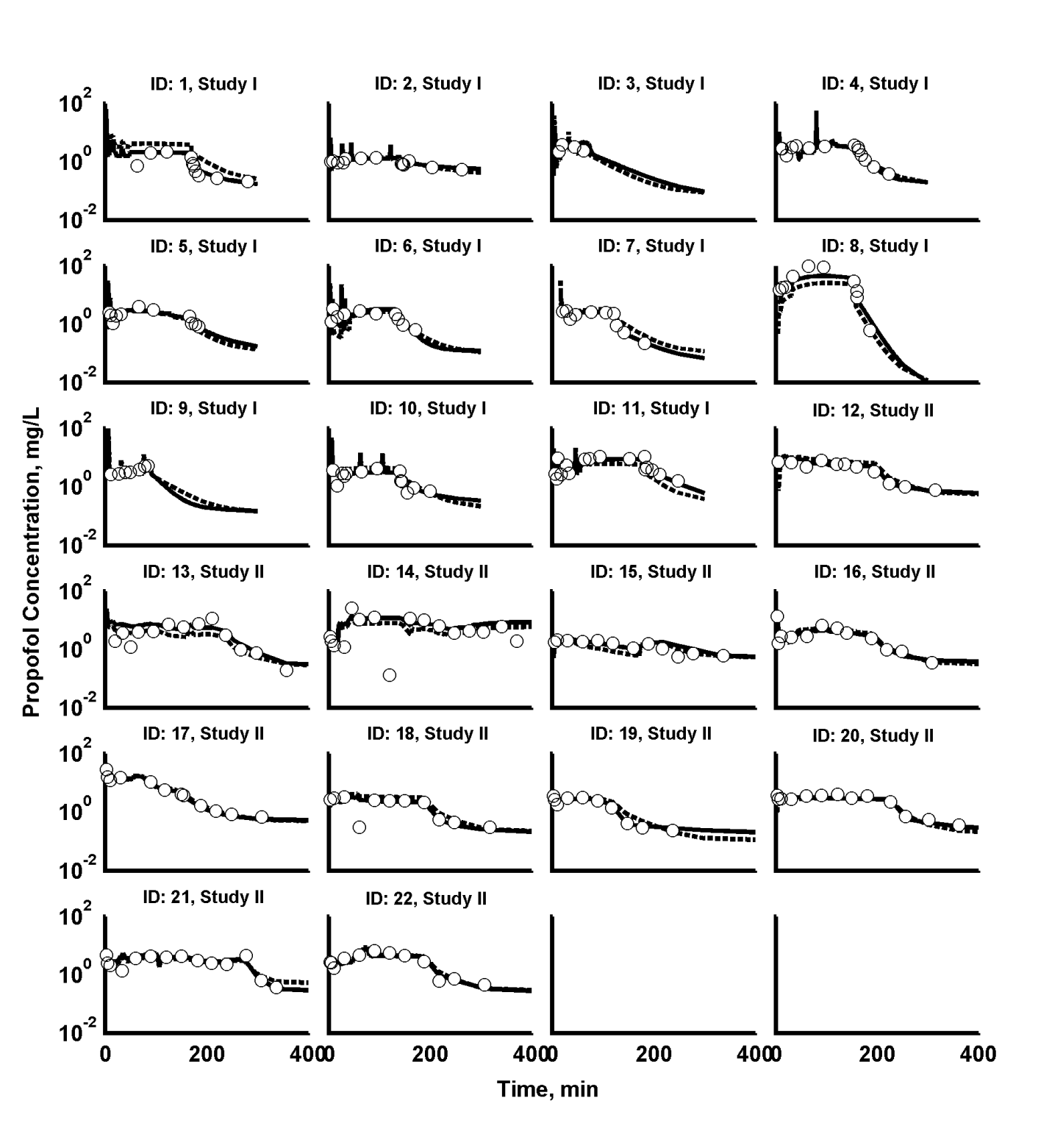


Figure 1S. Plot of observed (open circle), population predicted (dotted line) and individual predicted (solid line) propofol concentrations versus time for the final PK/PD model.


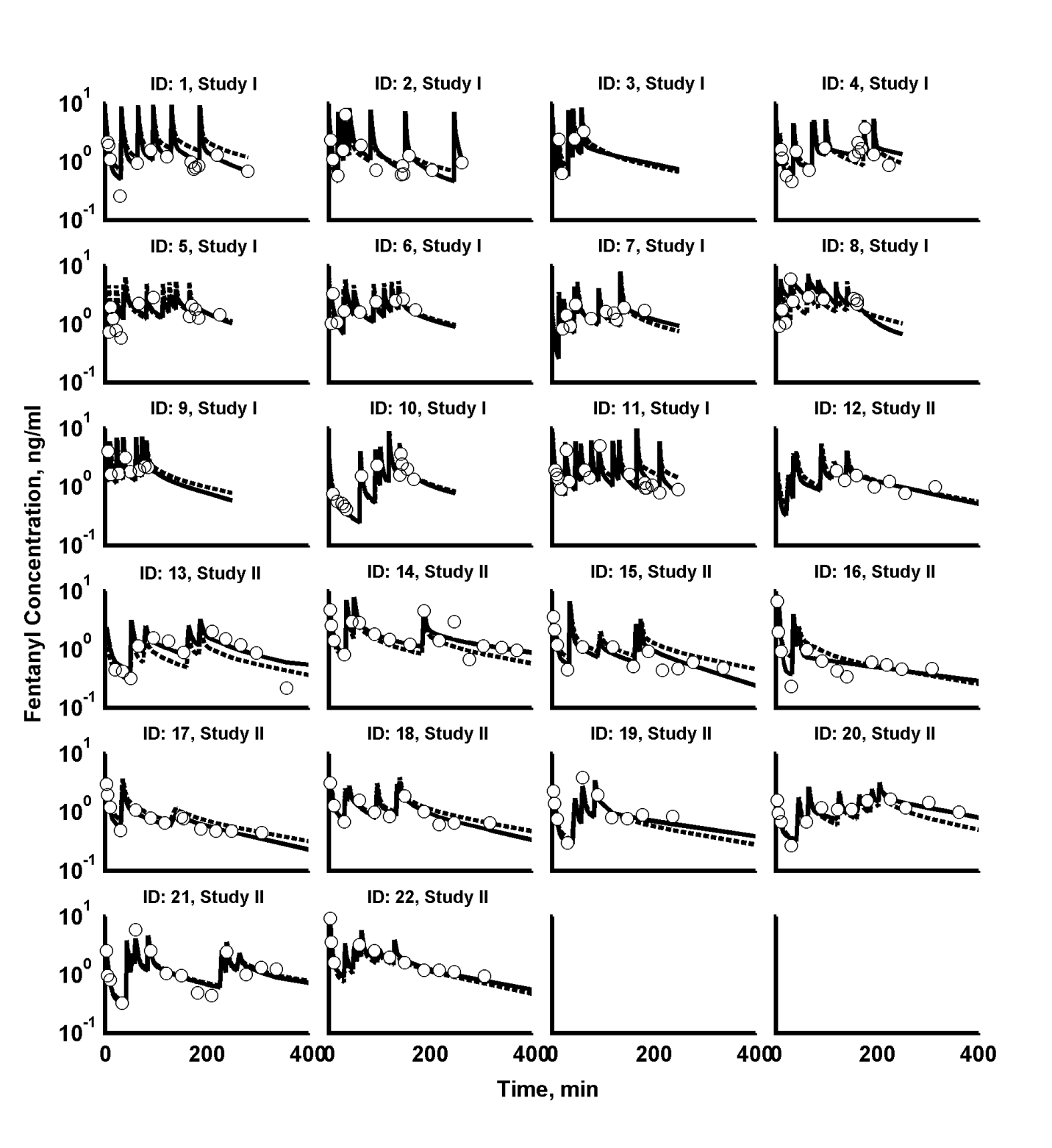


Figure 2S. Plot of observed (open circle), population predicted (dotted line) and individual predicted (solid line) fentanyl concentrations versus time for the final PK/PD model.


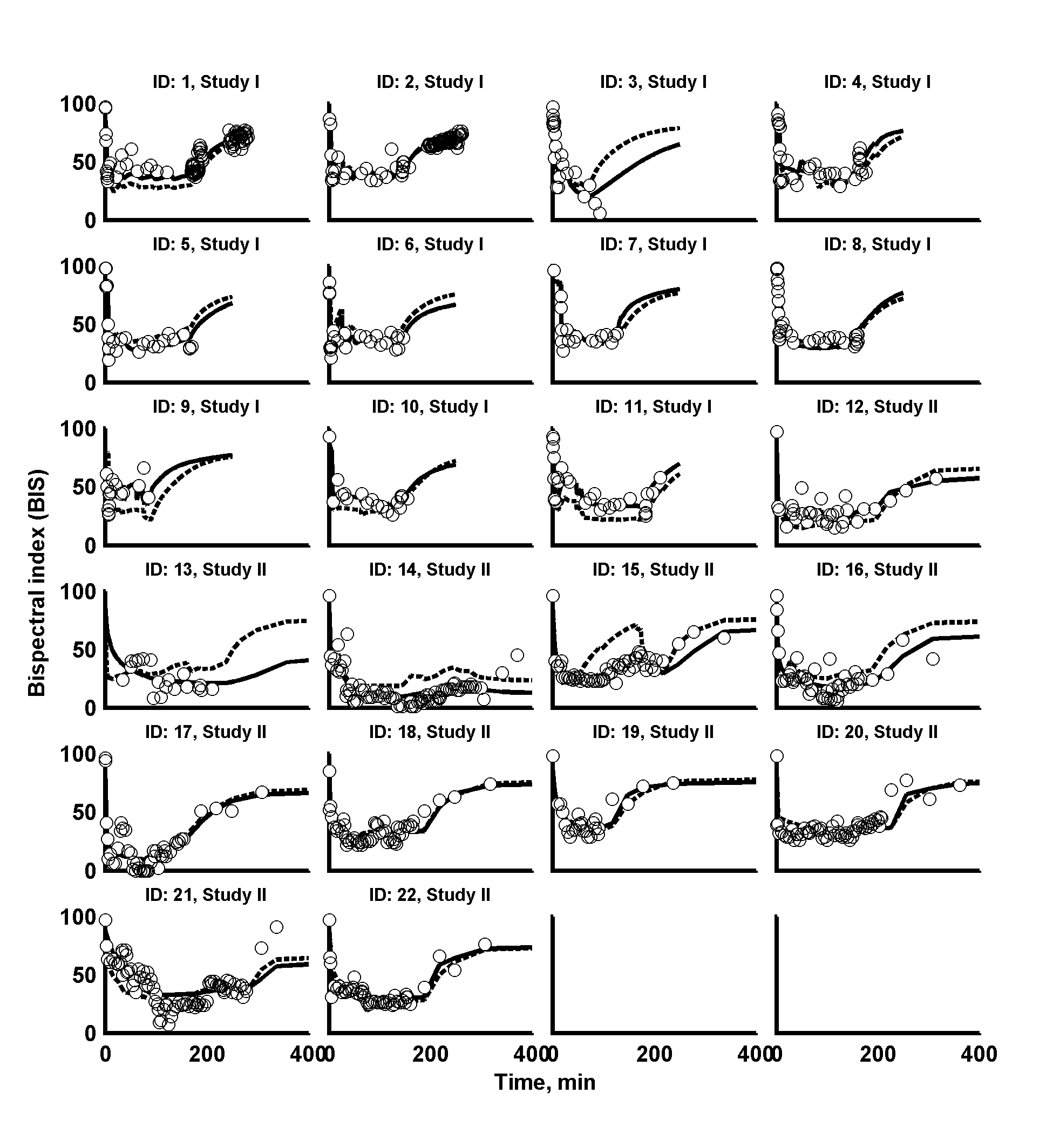


Figure 3S. Plot of observed (open circle), population predicted (dotted line) and individual predicted (solid line) BIS measurements versus time for the final PK/PD model.


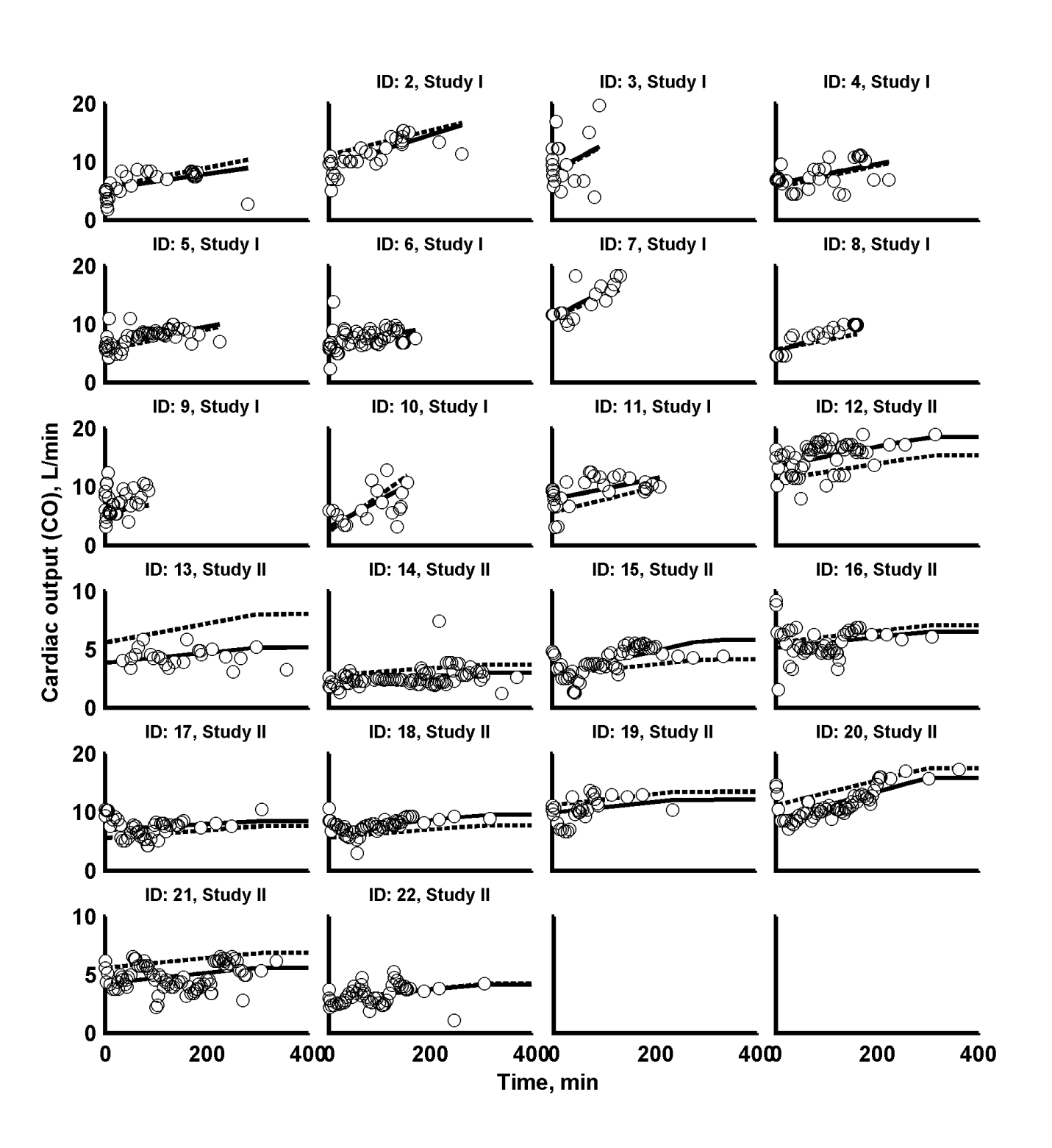


Figure 4S. Plot of observed (open circle), population predicted (dotted line) and individual predicted (solid line) CO measurements versus time for the final PK/PD model.


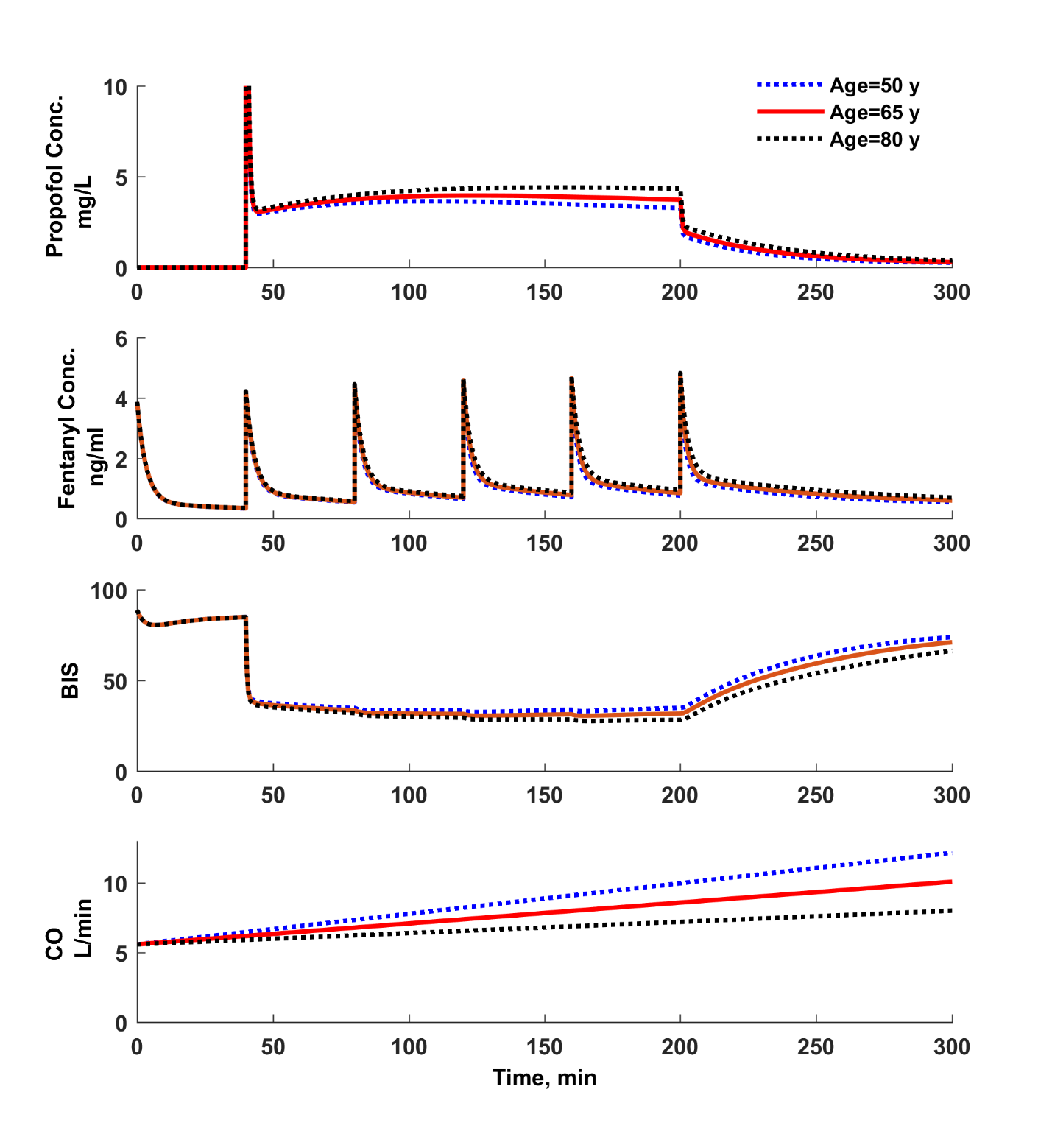
Figure 5S. The propofol concentration, fentanyl concentration, BIS index an cardiac output simulations from the final model for a study II subject of different age. The following infusion parameters were used: (propofol) initial dose 100 mg, rate of infusion 10 mg/min and duration of infusion 160 min, (fentanyl) multiple dose at 40 min intervals at a dose of 100 µg (solid line). The propofol infusion started 40 min after first fentanyl dose to illustrate the effect of fentanyl alone on BIS.


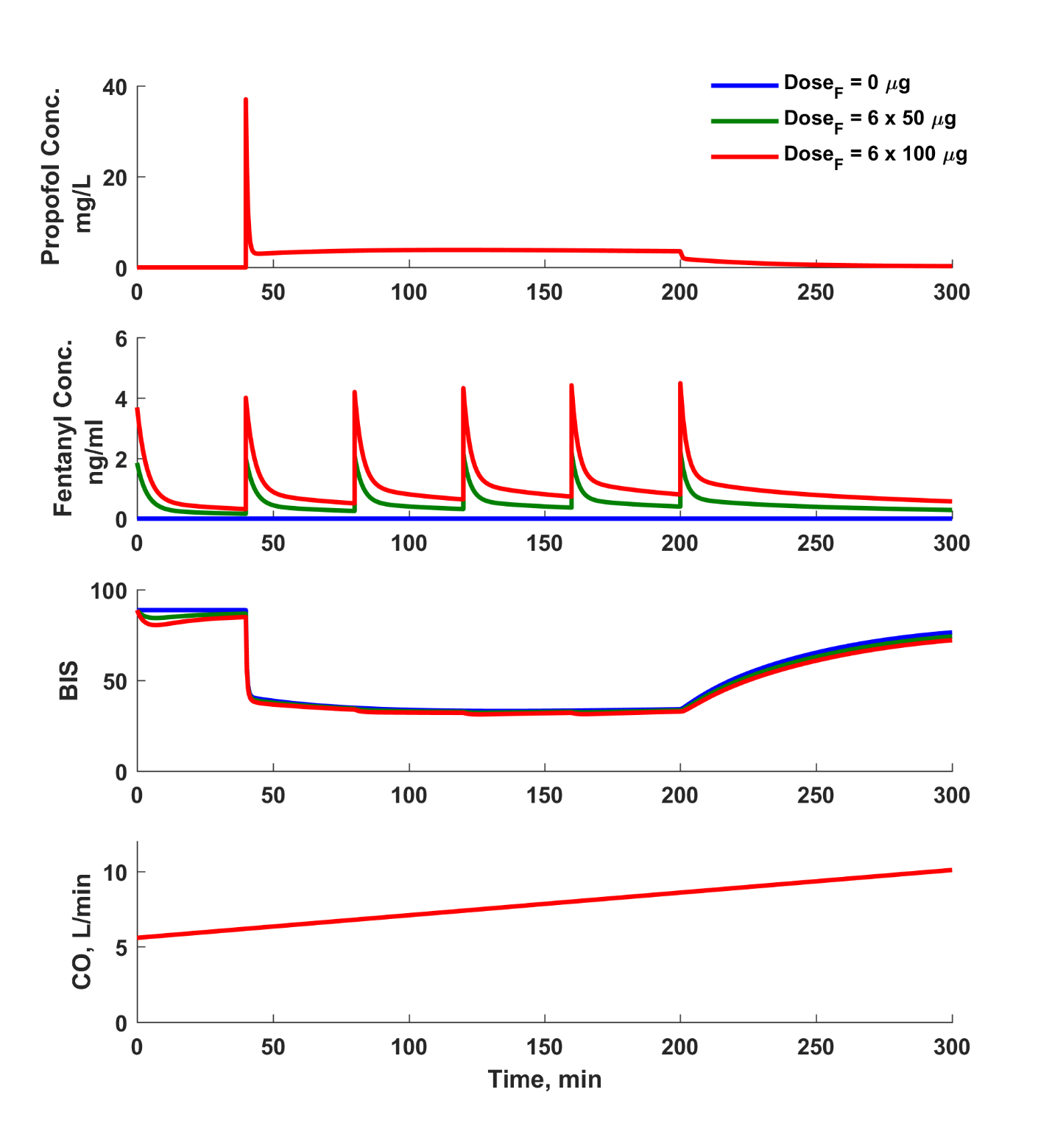


Figure 6S. The propofol concentration, fentanyl concentration, BIS index an cardiac output simulations from the final model for subject receiving different doses of fentanyl. Simulation are shown for a 65 years old subject from study II. The following infusion parameters were used: (propofol) initial dose 100 mg, rate of infusion 10 mg/min and duration of infusion 160 min, (fentanyl) multiple dose at 40 min intervals at a dose indicated in the graph.
